# More Technology, Better Learning Resources, Better Learning? Lessons from Adopting Virtual Microscopy in Undergraduate Medical Education

**DOI:** 10.1002/ase.1302

**Published:** 2012-08-28

**Authors:** Laura Helle, Markus Nivala, Pauliina Kronqvist

**Affiliations:** 1 Center for Learning Research, Department of Teacher Education, Faculty of Education, University of TurkuTurku, Finland; 2 Department of Pathology, Faculty of Medicine, University of TurkuTurku, Finland

**Keywords:** histology education, technology adoption, virtual microscopy, teaching of histology, computer-supported learning, medical education

## Abstract

The adoption of virtual microscopy at the University of Turku, Finland, created a unique real-world laboratory for exploring ways of reforming the learning environment. The purpose of this study was to evaluate the students' reactions and the impact of a set of measures designed to boost an experimental group's understanding of abnormal histology through an emphasis on knowledge of normal cells and tissues. The set of measures included (1) digital resources to review normal structures and an entrance examination for enforcement, (2) digital course slides highlighting normal and abnormal tissues, and (3) self-diagnostic quizzes. The performance of historical controls was used as a baseline, as previous students had never been exposed to the above-mentioned measures. The students' understanding of normal histology was assessed in the beginning of the module to determine the impact of the first set of measures, whereas that of abnormal histology was assessed at the end of the module to determine the impact of the whole set of measures. The students' reactions to the instructional measures were assessed by course evaluation data. Additionally, four students were interviewed. Results confirmed that the experimental group significantly outperformed the historical controls in understanding normal histology. The students held favorable opinions on the idea of emphasizing normal structures. However, with regards to abnormal histology, the historical controls outperformed the experimental group. In conclusion, allowing students access to high-quality digitized materials and boosting prerequisite skills are clearly not sufficient to boost final competence. Instead, the solution may lie in making students externally accountable for their learning throughout their training. Anat Sci Educ 6: 73–80. © 2012 American Association of Anatomists.

## INTRODUCTION

Digital tools have been argued to be among the drivers, or potential drivers, of curricular reform in medical education (Drake et al., 2009; Helle and Säljö, 2012). Histology education is a case in point. According to a recent survey conducted among allopathic and osteopathic medical schools in the United States, 44% of respondents indicated that they used virtual microscopy alone instead of glass slides, in comparison with only 14% in 2002 (Drake et al., 2009). The use of virtual microscopy is also being adopted in other regions of the world, although systematic international comparisons are hard to find. In his review of virtual pathology instruction, Dee (2009) reported that four of five Swiss medical schools, four of 12 medical schools in Poland, and three of 18 medical schools in Australia were using virtual microscopy. In addition, virtual microscopy is being used for distance learning in Canada as reported by Pinder et al. (2008).

Finland has been a leading force in developing technical solutions for virtual microscopy, such as the WebMicroscope (Lundin et al., 2004, 2009; WebMicroscope, 2012). As with other state-of-the-art virtual microscopy solutions, the WebMicroscope allows the user to manipulate a digital slide by zooming in and out of it on a computer screen while adjusting for brightness and contrast, as well as allowing the user to annotate the slide. The WebMicroscope was introduced to undergraduate students at the Faculty of Medicine of University of Turku, Finland, in 2008 as part of the pathology curriculum. The adoption of virtual microscopy created a unique “real-world laboratory” for exploring ways of reforming the learning environment with a series of instructional experiments (Helle et al., 2011; Nivala et al., 2012). The objective of the current study was to evaluate the impact of a set of measures (including digital resources) designed to boost understanding of abnormal histology through an emphasis on knowledge of normal cells and tissues. The performance of historical controls was used as a baseline, as previous students had never been exposed to the above-mentioned measures.

The manner in which undergraduate histology training is organized naturally depends in part on the curricular format of the medical school or faculty. In the traditional medical curriculum, normal structures are taught as a part of intensive courses of anatomy and abnormal structures as a part of pathology. This basic distinction can also be seen in integrated curricula; for instance, in the Dundee model, normal structure and abnormal structure belong to separate phases of the curriculum, although there is an explicit intention to build on former knowledge and skill (Harden et al., 1997). The results by Prince et al. (2003, 2005) indicated that curricular format per se does not appear to predict performance level in anatomy. Instead, Bergman et al. (2008) reported that performance appeared to be related to total teaching time in anatomy, teaching in a clinical context, and revisiting anatomy topics during the course of the curriculum.

Whether histology training is organized in the context of a traditional curriculum or an integrated curriculum, it tends to suffer from the fact that normal and abnormal histology are usually taught separately. First, from a practical point of view, it is problematic that some students enter undergraduate pathology training demonstrating a poor understanding of normal histology. Second, from a learning theoretical point of view, “simultaneous” exposure to a certain quality and a mutually exclusive quality (e.g., normal and abnormal) is considered highly conducive to perceptual learning (Marton and Pang, 2006). Therefore, the starting point of this field study was that perhaps the divide between normal and abnormal histology could be addressed with a set of measures applying modern information and communications technology (ICT), including virtual microscopy.

Many departments start adopting virtual microscopy simply by offering students “access to a collection of digital images.” Although this solution could be considered a student-friendly initiative, one may ask whether it is sufficient to promote learning. Some findings from nuclear medicine research suggest that this solution may contribute slightly to students' learning on the final examination (Heye et al., 2008). However, students might use these collections mainly to review for examinations, as page uploading peaks before examinations (Fónyad et al., 2010). Thus, whether offering access to digital collections of slides continuously promotes learning is unknown.

Virtual microscopy applications that allow for “highlighting structures, abnormal features, and normal areas for comparison” and inserting text lend themselves to creating integrated learning materials. Integrated learning materials serve to eliminate the need to look up information from separate sources before the material can be rendered intelligible. This type of material has been shown to speed up learning in diverse contexts (Ward and Sweller, 1990; Chandler and Sweller, 1992; Mayer et al., 1995). In addition, evidence suggests that virtual microscopy may also speed up initial content learning, as students' attention is not split between learning content and the intricacies of operating a light microscope (Husmann et al., 2009).

Surprisingly, few studies in the literature have investigated the use of ICT-based “course entrance examinations”; however, course entrance examinations do not appear uncommon in higher education. Gras-Marti et al. (2003) reported an impressive 30% increase in students' grades after an ICT-based course examination system was introduced for basic science courses. Taking the entrance examinations was voluntary, but the majority of the students chose to take the examinations. Furthermore, the students taking the examinations perceived the examinations positively. One may ask whether such an approach could be adopted with success in medical education.

Thus, the purpose of this study was to evaluate the impact of a set of measures (including digital resources) designed to boost understanding of abnormal histology through an emphasis on knowledge of normal cells and tissue structures. The research questions were the following: (1) How did the students react toward the elements of the intervention (normal review materials together with the course entrance examination, access to annotated digital slides, and virtual quizzes) based on the anonymous course evaluations? Based on the course evaluation data, whose needs were met by the elements of the intervention?; (2) Is there evidence that the intervention as a whole, or any of its elements, had an impact on the learning of histology?; and (3) How did the students use the available resources (based on interview reports)?

## MATERIALS AND METHODS

### Participants, Context, and Experimental Procedures

The participants (*N* = 126, 72 female and 54 male) were second-year medical students from the University of Turku, Finland, taking a basic pathology course in 2010/2011. The students (*n* = 105) completed an anonymous course evaluation form to tap perceptions of the learning environment and reactions to elements of the course. This information was complemented by information from 2009/2010 pertaining to one relevant item (evaluation of annotated virtual slides), which had been merged with another item in 2010/2011. In addition, 20 volunteers from 2010/2011 and 61 historical controls from 2007/2008 participated in assessments of histological knowledge (Helle et al., 2010). Furthermore, to gain insight into the use of the digital resources provided, four of the 20 volunteers from 2010/2011 were interviewed.

The context of the instructional intervention was a basic pathology course normally taught in the third semester (i.e., second year) of a six-year medical degree following a traditional curriculum. The course, as it was implemented in 2007/2008 and 2010/2011, consisted of a set of lectures, histology laboratory sessions, autopsy instruction, seminars, and examinations (see Table 1 for details). The instruction began with a set of lectures and histology laboratory sessions. The first three lectures and histology laboratory sessions dealt with general pathology (cellular injury, inflammation, and growth disorder), and the following 15 each dealt with different regions of organ pathology. The histology laboratory sessions were teacher-led, and during a period of nine weeks, a total of 200 slides representing a corresponding number of morbidities were studied. The students followed the instructor using a light microscope. The autopsy instruction was spread throughout the course. Once the lectures and histology laboratory sessions had ended, the students were expected to prepare for the practical microscopy examination and the final examination, which was focused primarily on biomedical understanding. The practical microscopy examination was identical in structure to the abnormal histology test, with the exception that it contained short questions pertaining to histopathology. The seminars were arranged toward the end of the course. Virtual slides were gradually introduced after the 2007/2008 term.

**Table 1:** Description of the “Healthy and Sick Human Being” Course at the University of Turku (2010–2011)^a^

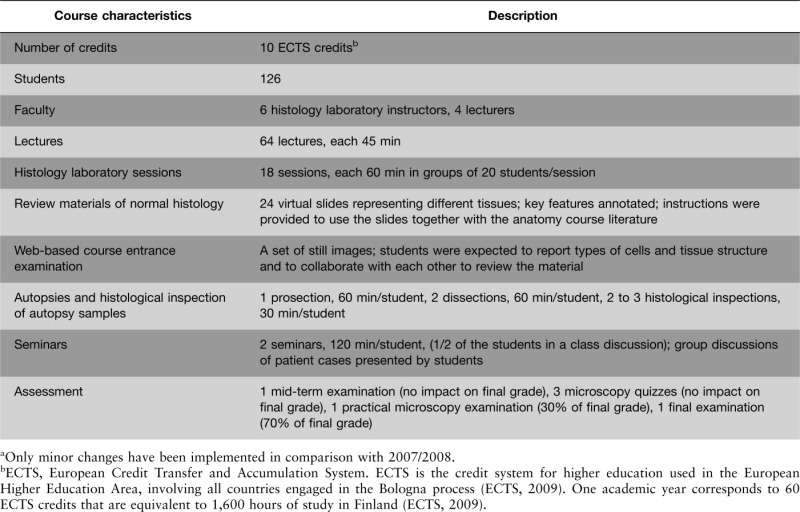

The experimental measures implemented with the WebMicroscope were as follows: (1) web-based materials to review normal cell and tissue structures, (2) a web-based entrance examination, (3) digitized slides with abnormal features indicated by graphics and text, with normal areas for comparison, and (4) three virtual quizzes taken in class for self-diagnostic purposes.

Before the start of the course, the students were asked to use web-based materials consisting of 24 annotated slides representing different types of normal tissue in conjunction with the anatomy course literature they had studied during the previous year. The purpose of the course entrance examination was to promote revision, as the students had already been taught normal structures in a systematic way during the first year of their studies. Therefore, this was not a strictly controlled examination; the students could choose when, where, and with whom they wished to take the test. They were asked to indicate cells and tissue structures present in a collection of still images. The entrance examinations were corrected by the teachers, and the students received their scores afterward. The three virtual quizzes were taken during the mandatory histology laboratory sessions. The students received immediate automated feedback (correct answer and number of correct answers in the class).

The research plan was approved by the Ethics Committee of the University of Turku.

### Materials and Data Collection Procedures

Anonymous course evaluation data were collected by the Faculty of Medicine for quality assurance purposes. The items from the course survey of 2010/2011 are presented in Table 2. These data were analyzed by zero-order correlations (Spearman ρ).

**Table 2:** Results of the 2010/2011 Students' Course Evaluation

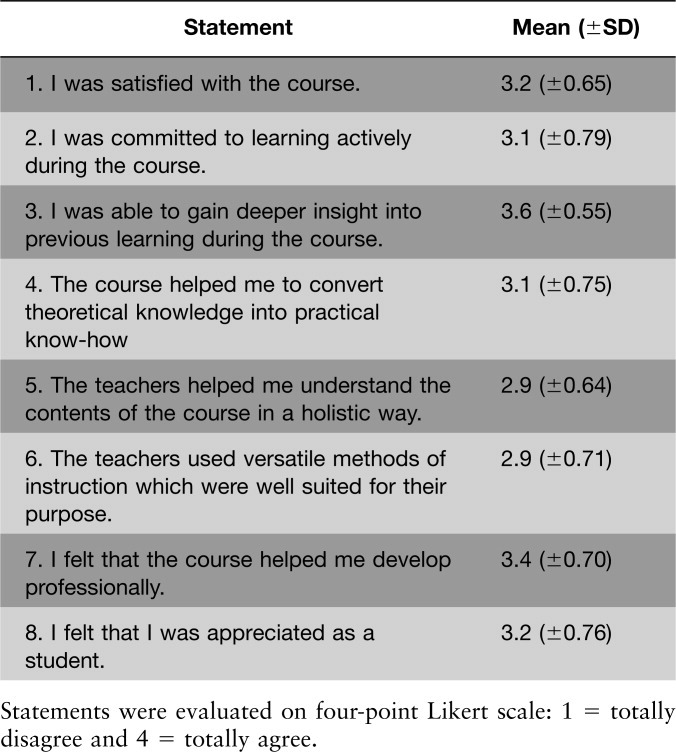

The students' performance in histology was measured twice to determine the short-term and longer-term impact of measures taken. Performance was measured at the beginning of the course (normal histology test) and near the end of the course (abnormal histology test). The students' performance was compared with that of historical controls. The students took the normal histology test approximately one week after the beginning of the course and the abnormal histology test one week before the practical microscopy examination. The tests (45 min each) were carried out during the students' spare time in a supervised computer classroom. Neither the tests nor the examinations included any slides from the study set.

In the normal histology test, the materials consisted of six authentic tissue samples presented using the WebMicroscope. For each image, the students were asked to (1) indicate the type of tissue, (2) identify and list as many cell types as possible, and (3) judge whether the sample looked normal or abnormal. The tissues presented were colon, lung, pancreas, breast gland, testicle, and skin. The answers for tissue type were rated by a senior pathologist on a scale from 0 = incorrect to 2 = correct. As for cell types, the students received one point for each correct answer. (No limit was set for the number of correctly identified cells.) For the normal versus abnormal question, the students received one point for each correct answer. As the reliability of the normal versus abnormal scale was very low and these items did not correlate with the whole, normal versus abnormal items were discarded from the test scale. The reliability for the scale containing cell type and tissue type items was 0.78.

The abnormal histology test consisted of a set of six tissue samples, from six authentic patients which did not overlap with the samples presented in the normal histology test. (The third sample for both groups was disqualified because it was not identical for the two groups.) In parallel with the samples, information about the samples' origins was presented. This time, for four of the samples, the participants were asked to (1) describe the abnormal histological features in the sample and to (2) suggest a diagnosis. For the two remaining samples, the diagnoses were given, and therefore, the participants were merely asked to list the key abnormalities. The test was scored by a senior pathologist. A point was given for each correct abnormal finding; however, four points was the maximum score for each case. Diagnoses were scored on a scale from 0 to 2, with “2” representing the complete correct answer. The reliability of the scale was 0.61.

To extract maximal information from the data, first-order questions pertaining to cells and features were treated separately from second-order questions pertaining to tissues and diagnoses. Independent sample *T*-tests were carried out separately for the normal histology and abnormal histology test with class (2007/2008 vs. 2010/2011) as the grouping variable. All results were checked with the Mann-Whitney *U*-test.

In addition, all of the students in the experimental group who had indicated on the informed consent form that they were available for a short interview were contacted. Four students were interviewed. Interviews took ∼15 min, were tape-recorded, and were transcribed verbatim. An outline of the interview is presented in the Appendix. The purpose of the interview was to answer research question 3 and to add student voices to the issues being addressed.

## RESULTS

### Students' Evaluations

**Figure 1:**
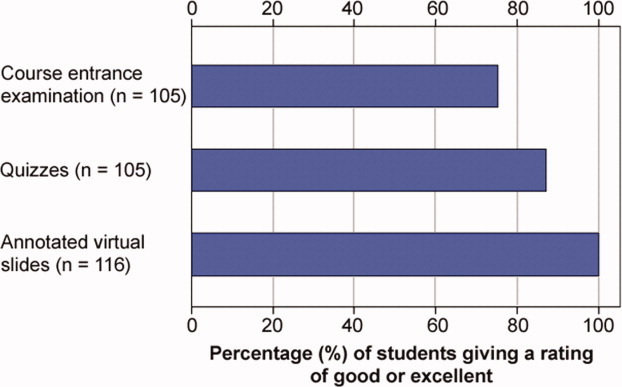
Students' evaluations of the elements of the intervention.

Students' evaluations to the course entrance examination, the virtual quizzes, and the annotated slides are presented in Figure 1.

As can be seen in Figure 1, the measures as a whole were relatively well received, as a clear majority of the students rated the measures “good” or “excellent.” The annotated slides were valued in particular: three of four indicated that they were excellent, and the rest indicated that they were good. Approximately one-fourth of the students found the course entrance exam “poor” or “fair.”

### Profile of Students Whose Needs Were Met

Based on the anonymous course data, the students who appreciated the virtual slides more than their peers had a distinct profile (Spearman ρ correlation with reaction to digital slides in parentheses): they expressed having been actively engaged in learning (ρ = 0.20); they expressed that they experienced the course as professionally relevant (ρ = 0.27); and they felt appreciated as students (ρ = 0.22). They also expressed appreciation for traditional study materials (ρ = 0.24) and for the versatility and appropriateness of the instructional methods as a whole (ρ = 0.25). The students' profile could be called “professionally motivated active learners.” Appreciation for the quizzes correlated with an appreciation for the mid-term examination (ρ = 0.43). This can be explained by the fact that both of these measures were self-diagnostic in nature: they did not have an impact on grades, but served as checkpoints. Appreciation for the entrance examination, however, was not related to any kind of student profile. It correlated only with the versatility and appropriateness of instructional methods (ρ = 0.21), a holistic approach in teaching (ρ = 0.24), and appreciation for the virtual quizzes (ρ = 0.41).

### Performance Measures: Experimental Group Versus Historical Controls

The results of the normal histology test and the abnormal histology test are presented in Table 3.

**Table 3:** Aggregated Scores of Items from the Normal and Abnormal Histology Tests

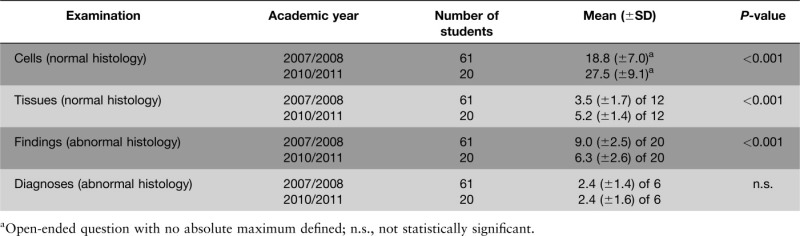

The results of the normal histology test taken one week after the beginning of the course indicated that students' understanding of histology increased in terms of both cell knowledge and tissue knowledge in comparison with the historical standard and that the increase was similar in magnitude for cell knowledge and tissue knowledge. Cell knowledge increased from *M* = 18.8 (SD = 7.0) to *M* = 27.5 (SD = 9.1) and tissue knowledge from *M* = 3.5 (SD = 1.7) to *M* = 5.2 (SD = 1.4), and the increase was statistically significant in both cases [*t*(79) = −4.43, *P* < 0.001; *t*(79) = −3.87, *P* < 0.001].

The results for the abnormal histology test taken one week before the practical microscopic examination revealed that, although the experimental group had scored higher on the normal histology test and had been exposed to a set of measures designed to promote learning, the experimental group scored “lower” on the abnormal histology test. The knowledge of findings decreased from *M* =9.0 (SD = 2.5) to *M* = 6.3 (SD = 2.6), and the decrease was statistically significant [*t*(79) = 4.18, *P* < 0.001], whereas the knowledge of diagnoses remained stable at *M* = 2.4 (SD = 1.4/1.6).

As attrition was high in measuring performance both in 2007/2008 and 2010/2011, an “analysis of attrition” was conducted. First, a test was performed of whether students participating in the study differed from their peers who did not participate in the study in terms of their grades in anatomy. It was found that among students in both the cohorts, those participating were slightly better achieving; however, there was no statistically significant difference. Second, a test was performed of whether the students participating in the study differed from their peers who did participate in the study in terms of their performance on the practical microscopy examination. It was found that among students in both cohorts, those participating in the study scored higher. This was especially the case in 2007/2008. Therefore, a rough estimate of the magnitude of selection bias was conducted by imputing the missing scores pertaining to findings on the basis of grades from the microscopy examination, as this was the only factor available correlating with abnormal histology test scores (neither gender nor the grade in anatomy correlated with performance on the test of microscopic pathology). The new set of results is presented in Table 4.

**Table 4:** Estimate of Aggregated Test Scores for Findings in Abnormal Histology with Imputed Missing Scores

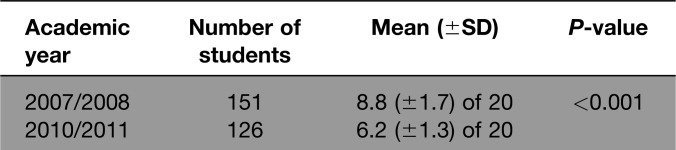

Table 4 shows that estimating the missing results by linear regression on the basis of the microscopy examinations of 2007/2008 and 2010/2011 had a minimal impact on the relation of performance between the two cohorts. This can be explained by the fact that the results of Table 3 were biased in the same direction in both the cohorts.

### Students' Accounts of Learning and Use of Virtual Resources

Students who were interviewed (*N* = 4) held a variety of opinions and perspectives. Students A and B were low achievers, based on their grades on the practical microscopy examination, whereas Student D was an average student and Student C was a high achiever. How did the interviewed students react to the basic idea of emphasizing normal structures? To put it briefly, the students embraced the idea. When asked, in the beginning, how it felt to study pathology during the course, Student D confessed: “Although there was a course during which you should have learned the normal samples, everything looked like pink porridge to me before [this course].” When explicitly asked about the use of annotated normal areas, Student D said: “I used them tremendously, because in the beginning of the course, my knowledge of normal samples was not that hot, and sometimes I came across a sample and I did not have a clue as to what it should look like normally, so it was good to have some confirmation without having to go to the trouble of locating a normal sample.”

In a similar vein, Student C, when asked about reviewing normal structures before moving on to abnormal ones, pointed out: “Extremely useful to gain a touch for [the normal]… or you remembered what healthy looks like, and you had to review it again during the course, kind of like compare, because disease does not always have the appearance of disease. A very good thing, indeed.” Students A and B were less verbose, but agreed that reviewing the normal tissue was very useful.

However, the extent to which the students prepared for the entrance examination and used the digital resources appeared to vary significantly. Two of the students reported having spent one or two hours preparing for the entrance examination. Student D reported having taken the examination in a group and merely taking a look at the materials. Student A reported having spent an hour or two in preparing for it. The other two students admitted having invested more time: Student C estimated he had prepared for a maximum of five hours. Student B was unable to provide an estimate, but reported having prepared “a lot,” and also used other resources. Student A admitted he hardly used the digital slides before he reviewed for the examination. At the other end of the spectrum, Student B, who did not have a background in medicine but had knowledge from a related domain, reported having made considerable use of the learning resources. She reported having spent one to two hours inspecting the slides before each histology laboratory session. Student C who, based on objective measures made the most rapid progress, reported having inspected the slides for more than half of the histology laboratory sessions (approximately a half hour of viewing per histology laboratory session). In a similar vein, Student D reported having tried to study the slides “with varying amounts of success” for approximately half an hour.

The students also reported different ways of making use of the annotated slides. In part, the strategy depended on whether the student was reviewing for the examination or for a histology laboratory session. In preparing for these sessions, Student D reported not restricting himself to the annotated areas: he reported trying to locate features indicated by the annotations “outside” the annotated areas. Student B reported reading the adjacent text, then studying the general picture and the annotated areas before the histology laboratory sessions. Student C reported that it was good to be able to review at home before the histology laboratory sessions and pointed out that there were basically two options: to view without the annotations in search of lesions or to examine the annotated areas directly, “which in part, perhaps, makes it too easy, although I found it extremely good in any case.” Student D relied on the written course worksheets prepared by the teachers. He first studied the description in the worksheet, then the annotations, and then tried to find some abnormalities outside the annotated areas. Student A reported using the slides only to review for the examination. Two students (A and B) reported that they prepared for the examination by hiding the annotations.

## DISCUSSION

The results indicated that the experimental group exposed to web-based review materials and a course entrance examination outperformed historical controls on the normal histology test taken a week after the beginning of the course. This result was obtained despite the fact that the entrance examination was resented by one of four students taking it, and the pattern of attrition was in favor of the historical controls. However, on the abnormal histology test taken a week before the practical microscopy examination, the experimental group was outperformed by the historical controls. The interviews confirmed that the reported use of the annotated slides varied considerably.

These results suggest that reviewing of normal histology before the course and taking the web-based course entrance examination boosted knowledge of histology in the “short term.” However, boosting prerequisite skills and allowing students access to high-quality virtual materials did not appear sufficient to boost learning continuously. Second, the results suggest that students' performance may be improved when students are externally accountable for their performance. The self-assessment measures taken during the instructional period appeared insufficient for maintaining learning efforts by historical standards.

The main limitation of this study is the high attrition rate pertaining to the performance measures. The analysis of attrition revealed that the pattern of attrition favored the historical controls. However, based on the authors' calculations, this is unlikely to have been the sole cause of the difference between the experimental group and historical controls on the abnormal histology test. In this type of design, cohort effects are also possible. For this reason, the teaching instructor was asked if there was anything that could have led to different results in 2007/2008 and 2010/2011. She pointed out three things: (1) the students in 2010/2011 had two fewer histology laboratory sessions (general cytology and gynecological cytology were excluded), (2) the instructors teaching the histology laboratory sessions may have changed, and (3) the student cohort in 2010/2011 appeared a bit passive, as reflected by the participation rate on an elective course in pathology containing three hours of microscopy. The first explanation can be ruled out, as the abnormal histology test did not contain cytology samples. Although it cannot be ruled out that the 2007/2008 students may have been exposed to slightly better teachers, it must be emphasized that the teachers were all equally qualified people, and the teaching style used was uniform. More importantly, some evidence based on course evaluation data was found suggesting that, during the time period from 2006 to 2009, students reported perceiving pathology as progressively less relevant from a professional point of view. In 2010, the evaluators adopted a new evaluation framework, and therefore, the evidence on this issue is not conclusive.

Even if the factors listed above accounted for the “difference” in the abnormal histology test in favor of the control subjects, how does one account for the apparent lack of impact of the intervention? The obvious explanation, confirmed by the interviews, is that the measures taken are not sufficient to induce all of the students to actually access the slides more than a few days before the practical microscopy examination. Furthermore, providing automated feedback of students' performance, as in the case of the quizzes, may not be an effective strategy, as indicated by the results by Wieling and Hofman (2010). In some cases reported in the literature, introduction of digital resources has even led to negative effects, such as decreased attendance (Traphagan et al., 2010) and procrastination in learning efforts. For instance, Collier et al. (2012) reported that the introduction of virtual slides led students to procrastinate in learning histology. The authors interviewed teaching assistants taking a basic course in anatomy for premedical students. To the concern of the teaching assistants, the introduction of virtual slides led students to focus on gross anatomy during laboratory sessions, as the students evidently thought that they could more easily learn the histology on their own at home. The suggested solution was to arrange laboratory sessions focusing solely on histology.

An alternative (or complementary) explanation is that relatively good performance in the beginning induced a sense of security, which may have decreased the pressure to continuously review materials. In fact, a study by Ahopelto et al. (2011) indicated that students with a moderate initial understanding appeared resistant to learning, leading the investigators to interpret that “an illusion of understanding” may have come into play.

Before moving on, the authors would like to emphasize that they are in no way skeptical about either the potential or the value of introducing virtual microscopy. (For a skeptical note on the use of ICT in higher education, see Kulesza et al., 2010.) On the contrary, virtual microscopy opens up unprecedented possibilities for modernizing the teaching of histology. The authors' point, based on the existing data, is that there is no guarantee that the affordances of a technology are transformed into actual learning. The adopting of such a technology may bear with it some rather unexpected consequences due to the human factor, and these consequences may require further action. Based on the literature, success in technology adoption is best achieved through a process of sustained collaboration between the evaluators and the developers (Leonard, 2004; Sheeby et al., 2006).

There are various directions that may be taken for future research. The next version of WebMicroscope allows user-specific data on actual page loading to be collected. This will give investigators more detailed information on the patterns of usage to corroborate students' self-reports. The difficulties inherent in comparing samples from different cohorts may be avoided by exploring different ways of adopting web-based learning directly with crossover designs (Cook et al., 2008).

## CONCLUSIONS

Arguably, with the annotated, digital materials, teachers have created a more flexible, more student-friendly learning environment. However, without the proper incentives, students may fail to make the fullest use of virtual microscopy. The solution may lie in making students externally accountable for their learning throughout the training period. In fact, Wiliam and Thompson (2007) argue that to integrate assessment with learning effectively, the teacher must assume the role of engineering effective classroom discussions and tasks that elicit evidence of students' learning (and in which students can serve as instructional resources for one another). As far as the authors see, this can be achieved in several ways: (1) more class time should be used for discussion on patient cases; (2) the students can submit project reports or other assignments, which are graded, and for which the students receive feedback; the results of Helle et al. (2011) indicated the feasibility of this approach for students with sufficient background knowledge for independent work; and finally, (3) more class time in histology laboratories can be reserved for active engagement with materials and staff (McBride and Prayson, 2008). The caveat is that such activities cannot be incorporated without reducing the amount of material that the teachers cover. However, given modern digital resources, why, for instance, do all of the course slides have to be covered in the histology laboratory sessions? Given that the objective is to understand basic disease processes, could the students work more in depth with perhaps fewer slides?

## APPENDIX

Interview questions.

1. How did it feel to study pathology as part of the course “Healthy and Sick Human Beings”?
2. How would you evaluate the resources you invested in studying during the course on a scale from 1 to 5? How did it show? Did you invest more or less than usual? Why?
3. Could you give me an estimate of the number of hours that you put into studying for the course entrance examination? How useful did you consider reviewing healthy structures before moving on to abnormal ones? What is your opinion about having a course entrance examination?
4. Could you give me an estimate of how much you used WebMicroscope on your own time (in hours)? Did you use it throughout the course or during a specific period? How was its use positioned in relation to the histology laboratory sessions? Did WebMicroscope facilitate or in some way impede your studying? How? Did you also practice with a light microscope? If so, for how much time?
5. How did you use WebMicroscope? Did you inspect the virtual slides on your own or together with others? To what extent? Did you make use of the possibility to view normal and abnormal areas at the same time?
6. How would you evaluate the quality of the materials (virtual slides and materials for the entrance examination)?
7. How useful do you consider practicing microscopy from a professional point of view? Why? Did your motivation affect your performance in the course? How?
8. Can you think of ways to improve the course/instruction?
9. Do you have something you would like to communicate to the researchers?
